# Zinc Deficiency—An Independent Risk Factor in the Pathogenesis of Haemorrhagic Stroke?

**DOI:** 10.3390/nu12113548

**Published:** 2020-11-19

**Authors:** Kurt Grüngreiff, Thomas Gottstein, Dirk Reinhold

**Affiliations:** 1 Clinic of Gastroenterology, City Hospital Magdeburg GmbH, 39130 Magdeburg, Germany; thomas.gottstein@klinikum-magdeburg.de; 2 Institute of Molecular and Clinical Immunology, Otto-von-Guericke University, 39120 Magdeburg, Germany; dirk.reinhold@med.ovgu.de

**Keywords:** zinc resorption, zinc functions, food composition, zinc deficiency

## Abstract

Zinc is an essential trace element for human health and plays a fundamental role in metabolic, immunological and many other biological processes. The effects of zinc are based on the intra- and extracellular regulatory function of the zinc ion (Zn^2+^) and its interactions with proteins. The regulation of cellular zinc homeostasis takes place via a complex network of metal transporters and buffering systems that react to changes in the availability of zinc in nutrition, chronic diseases, infections and many other processes. Zinc deficiency is associated with impairment of numerous metabolic processes, reduced resistance to infections due to impaired immune functions, changes in skin and its appendages and disorders of wound healing and haemostasis. While ischemic heart attacks (myocardial infarction) occur more frequently with meat-based normal diets, haemorrhagic strokes are more frequently observed with vegetarian/vegan diets. The causes are discussed as deficiencies of various micronutrients, such as vitamin B12, vitamin D, various amino acids and also zinc. In the present review, after a description of the functions of zinc and its resorption, a discussion of daily food intake will follow, with a special focus on the importance of food composition and preparation for the zinc balance. The close interrelationships between proteins, especially albumin and zinc will be discussed. Finally, the possible causes and consequences of a zinc deficiency on the blood vessels and blood coagulation are considered.

## 1. Introduction

The results of a prospective epidemiological observational study [1], including 48,188 participants over 18 years of age, on the occurrence of ischaemic heart attacks and strokes with different diets show that a vegetarian/vegan diet is not, per se, the better dietary form in every situation compared to a mixed, meat-based diet.

While the more frequent ischaemic heart attacks with a meat-based normal diet are more in line with the assumptions and experiences known so far, the increased incidence of strokes, especially the increased incidence of haemorrhagic strokes in vegetarians and vegans is surprising. As possible causes, the authors discuss among other things: “lower circulating levels of nutrients vitamin B12, vitamin D, amino acids and long-chain *n*-3 polyunsaturated fatty acids.” From our point of view and experience, deficiencies of trace elements such as zinc and iron are also to be considered as further possible factors in this context.

## 2. Functions of Zinc

Zinc is an essential trace element that intervenes in a variety of metabolic processes and thus plays a fundamental role in human health. The biological effects are based on the intra- and extracellular regulatory functions of the zinc ion (Zn^2+^) and its interactions with proteins [2]. The ion is an important component for the catalytic activity of more than 300 enzymes, exerts structural effects on various transcription factors and regulates hormones, hormone receptors and gene expression [3,4,5]. Moreover, it is enzymatic cofactor in the regulation of carbohydrate, fat and protein metabolism and plays an important role as a second messenger, as a signal ion, it has an antioxidant effect and influences the redox metabolism, although the zinc ion (Zn^2+^) is redox inert [3].

Zinc is essential for innate and acquired immunity and for the regulation of numerous reactions in haemostasis and thrombosis [6,7]. It is of great importance for platelet aggregation and fibrin formation, activation of the contact system on artificial surfaces, interactions between the contact system and the endothelium (regulation of thrombosis), as well as for coagulation, anticoagulation and fibrinolysis [7,8].

Thus, this trace element plays an important role in the organisation and regulation of several physiological and pathophysiological processes, like wound healing, membrane repair, oxidative stress, coagulation, inflammation and immune defence, tissue re-epithelialisation and other [7].

Furthermore, it belongs to the group of type 2 nutrients (e.g., nitrogen, essential amino acids, proteins, albumin, magnesium and potassium). In contrast to type 1 nutrients such as iron, thiamine, niacin, vitamin C and folic acid, which have few specific functions and whose deficiency leads to a specific metabolic disorder, type 2 nutrients are important for numerous metabolic processes [5]. Insufficient uptake or disease-related loss of type 2 nutrients leads to a marked reduction in excretory elimination in order to avoid deficiency, especially in the case of high-demand and very important functions such as growth and immunity [5].

Due to the very close relationship between serum albumin and serum zinc with a molar ratio of 30:1 kept within narrow limits, changes in one factor is accompanied always by similar changes in the other. A decrease in albumin concentrations, for example, in inflammatory situations, is accompanied by a decrease in zinc concentrations. On the other hand, a decrease in blood concentrations of zinc leads to a change in protein metabolism with a reduction in urinary nitrogen excretion, reduced concentrations of prealbumin and albumin in serum and of retinol-binding protein [9]. With these close interrelationships of the type 2 nutrients zinc and albumin, reduced protein intake in animal-based diets also causes a reduced zinc intake [9].

Recent investigations by Coverdale et al. [10] confirm and expand the knowledge on the impact of elevated concentrations of free fatty acids (FFAs) on the interactions between proteins, especially albumin and zinc. Physiologically relevant long-chain FFAs, for example, palmitate and stearate, have a higher affinity to albumin than zinc. The authors conclude that a reduced binding capacity of albumin to zinc at elevated concentrations of FFAs leads to zinc redistribution and thus significantly influences physiological and pathological processes.

These diverse metabolic effects and the numerous protein bindings are the main reasons for the difficulties in finding a specific biomarker of zinc supply similar to ferritin and transferrin in iron [5]. Possible starting points for the identification of a specific biomarker for changes in the cellular zinc pool could result from measurable changes in the zinc transporters responsible for zinc homeostasis and in metallothionein (MT) [5].

## 3. Resorption of Zinc

Almost 95% of zinc is located intracellularly so that the extracellular concentration available is only low. Circulatory zinc is mainly bound to albumin, transferrin and α2-macroglobulin but remains accessible to zinc transporters to control the cellular zinc balance [11].

In addition to gender and age, the time of food intake and blood sampling are of particular importance for the determination of serum/plasma zinc concentrations (0.1% of total body zinc), which exhibit circadian variations [12,13,14,15]. The decrease in blood zinc concentration begins 0.5–1.5 h after meals. The lowest levels are reached about 3–4 h after eating [15]. Women usually have lower zinc levels than men due to lower body weight and muscle mass [15]. If possible, the necessary blood samples should be taken in the morning from the sober patients.

Organs with high zinc content are the liver, muscles, pancreas, testicles, prostate and the retina of the eye [16]. In contrast, the cellular zinc concentration is relatively high [2,5,17]. There are close interactions between these two compartments. Plasma zinc is a component of the so-called small, vulnerable pool and a clinically reproducible marker of zinc supply [5].

The zinc metabolism in higher eukaryocytes is complex and there is control of uptake, release (efflux) and storage in individual cells, both in peripheral tissues and organs [18]. In the last two decades, there has been great progress in understanding the genes involved in these processes and their regulation [18]. For example, the metal-response element-binding transcription factor-1 (MTF-1) is a cellular zinc sensor that coordinates gene expression, zinc homeostasis, protection against metal toxicity and oxidative stress and embryonic development [18].

All cells need a constant zinc supply in order to maintain the cellular zinc homeostasis, which is indispensable for the fulfilment of the manifold cell functions [19]. This is achieved by cooperative action of 24 zinc transport proteins (ZIP, SLC39-Influx and ZnT SLC30-Efflux) [20,21] and various intra- and extracellular zinc-binding proteins, such as MT (intracellular) as well as albumin, alpha-macroglobulin, transferrin and calprotectin (extracellular) [19].

The absorption of zinc occurs in the small intestine, especially in the jejunum. Zinc uptake is realised by two different mechanisms: (i) by a saturated, carrier-mediated process and (ii) by a non-mediated, passive process [22]. The intestinal excretion of endogenous zinc is dependent on the current absorption and zinc status as reaction and response [11,23].

## 4. Zinc and Nutrition (Diet)

A healthy person loses 2–3 mg zinc daily [24,25]. The daily zinc requirement of healthy adults is 7–11 mg (for children, aged 1–10 years: 3–7 mg), where the recommended intakes vary considerably from country to country [25]. During an illness but also under heavy physical strain due to work or sport, the excretion of zinc via faeces, urine, sweat and skin can vary greatly from a healthy adult. An increased zinc requirement (10–15 mg) also exists during pregnancy [4].

When assessing the dietary zinc supply to humans, it is important to remember that the zinc content of a foodstuff (nutrients) is not an indicator of its bioavailability. There is a big difference between the zinc content of a food and its availability. Some cereals contain sufficient zinc but it is not available because it is localised in very specific regions of the seed grain, which are not released during milling [26]. For example, the availability of zinc in peas, lentils and beans is limited [26,27].

Most zinc is found in oysters and wheat germ, although bioavailability from wheat germ is also limited, followed by muscle meat and animal offal and by far potatoes and wholemeal bread [27]. Leafy vegetables and fruits, in particular, have low zinc concentrations with high water content [16,28,29]. Since meat contains a lot of zinc, meat-based mixed diets can hardly cause zinc deficiency in healthy people due to their diet. It should be noted that red meat (beef) contains more zinc than white meat (e.g., chicken) and also fish. On the other hand, vegetarian but especially vegan nutrition is often associated with a zinc deficiency, if the recommended foods with a higher zinc content are not used, such as whole-grain products, tofu, soy products, oat flakes, brown rice and nuts, as plants contain significantly less zinc [28]. In addition, food components impair the absorption of zinc. In healthy people, the absorption rate is 20–30% [30]. The zinc content of plants is also influenced by the zinc concentration in the soil [22]. The physiological functions of zinc depend on its bioavailability in cells and tissues, which in turn is closely related to intestinal absorption [31].

The main inhibitor of intestinal zinc absorption is phytate, which is found in unrefined cereals, pulses, oilseeds and nuts. Fruits, roots, tubers and leafy vegetables contain little phytic acid. Phytate forms insoluble complexes with zinc in the intestine, which hinder the absorption and bioavailability of zinc [23,24,25,32]. The consequence is reduced zinc absorption in the intestinal cells [19]. Further processing such as grinding, soaking, germination, malting or fermentation can reduce the phytate content of foodstuffs and thus the inhibitory effect [28].

Various amino acids such as cysteine and methionine, which are found in grains, nuts, cereals and vegetables but also histidine and organic acids such as citric acid (citrus fruits), lactic acid (sour milk) as well as fruit acid, bind zinc and increase absorption [25,28].

Proteins influence zinc absorption in different ways [28]. While casein in milk has an inhibitory effect on zinc absorption, soya, once the phytate has been removed by precipitation, no longer influences the absorption of zinc [25,28]. Vegetarians adapt to the lower zinc intake after some time by increased absorption and retention of zinc. An impairment of zinc resorption by the iron contained in the diet is only to be expected, even with the addition of iron, if the iron-zinc ratio is very high [25,27].

Other food components, which reduce zinc resorption or bioavailability, are fibres and higher calcium concentrations [33]. It is very likely that the absorption-inhibiting effect of these substrates is also due to the phytate they contain. Studies on isolated fibre components, for example, alpha-cellulose, do not show an inhibitory effect on zinc absorption [34]. Fortification of a diet low in meat does not increase the rate of zinc absorption because the addition of minerals slightly reduces zinc resorption [33,35].

The absorption of zinc from supplements appears to be higher than from food because there are no significant inhibitory factors [36]. However, the benefit of multi-mineral supplements is again limited due to their instability and possible irritation of the small intestinal mucosa [31]. According to recent experimental data, the bioavailability of zinc but also of other substrates, can be significantly improved by “food-derived zinc-chelating peptides” containing cysteine, histidine, serine, aspartate and glutamate. However, their effectiveness still needs to be tested in large clinical trials [31]. When taking zinc supplements, the occurrence of possible interactions with drugs such as antibiotics (e.g., ciprofloxacin, tetracycline), diuretics (e.g., thiazides) and penicillamine must also be taken into account [37]. Compared to those on a mixed diet, vegetarians are recommended to consume more zinc (up to 150%) with their diet [28,33].

Vegetarians and individuals with a low protein intake of animal origin have lower zinc scores and a higher ratio between phytate and zinc, based on the data of a biochemically validated questionnaire for determining human zinc status [32].

In a one-year study on the behaviour of plasma concentrations of zinc when switching from a mixed meat-based diet to a vegetarian diet, both plasma and urinary zinc levels decreased after three months but did not decrease further after six months [38]. Foods with a phytate-zinc molar ratio of >15 generally have low zinc bioavailability, those with a molar ratio of <5 have good zinc bioavailability [22]. In a World Health Organisation (WHO) report [39], a lacto- and ovo-vegetarian diet as well as a vegan diet with a phytate-zinc molar ratio of 5–15 is evaluated as moderately bioavailable (30–35% absorption rate). Diets with high bioavailability of zinc (50–55% absorption) have a molar phytate-zinc ratio of <5. They contain mainly refined cereals, few fibres and meat-based adequate protein intake [33].

## 5. Zinc Deficiency

In developing countries, more than 25% of the population suffers from zinc deficiency due to inadequate zinc intake, while in industrialised countries the figure is as high as 15% [19]. Populations at greatest risk of inadequate zinc intake in industrialised countries are pre-school children, elderly people as well as vegetarians and vegans, who all eat lower amounts of meat-based foods [40].

As mentioned above, no laboratory method or biomarker for the exact definition of a zinc deficiency exists to date. The determination of zinc concentrations in serum or plasma with defined trace element-free collection systems is considered a reliable measure that is well able to be reproduced in everyday clinical practice.

The term “zinc deficiency” describes a reduction of the zinc levels in serum or plasma, combined with corresponding clinical symptoms. Measurement in these two compartments is the only indicator recommended by the World Health Organisation (WHO), UNICEF and other organisations for estimating the zinc status in the population [40,41]. Marginal zinc deficiency is usually not associated with functional or biochemical disorders. In contrast, severe zinc deficiency quickly leads to metabolic changes. The zinc balance becomes negative with a net loss of zinc from the small, rapidly replaceable pool [40]. Unlike many other nutrients, zinc has no functional reserve or body stores of available zinc [40].

Besides an insufficient zinc intake with food, genetic causes, chronic alcohol consumption and also various medications (corticoids, contraceptives, etc.), zinc deficiency can occur in the course of various diseases. These include chronic liver, pancreas, kidney and chronic inflammatory intestinal diseases, diabetes mellitus, collagenosis, especially rheumatoid arthritis but also tumour diseases, myocardial infarction and infections [42].

In addition to the synthesis of albumin and other proteins, zinc deficiency also impairs haemoglobin formation (Hb) [15,43]. The synthesis of Hb is controlled by various zinc enzymes, such as delta aminolevulonic acid synthetase, thymidine kinase and DNA polymerase [44,45,46]. In addition, zinc is important for stabilising red blood cell membranes and maintaining effective plasma IGF-1 levels to stimulate erythropoiesis [45].

Clinical signs of prolonged zinc deficiency are disorders of the sense of smell and taste, dark adaptation, changes in the skin and its appendages (brittle nails, dry, scaly skin, wound healing disorders, increased susceptibility to infection due to a disturbed immune system and cerebral dysfunctions [41].

## 6. Zinc Deficiency: Influence on Vessels, Coagulation and Stroke

Zinc is essential for endothelial integrity [24]. Zinc deficiency leads to severe damage to the endothelial protective function and causes or enhances a cytokine-mediated inflammatory process [47,48]. Henning and colleagues [49] were able to prove in experimental studies that the addition of zinc in zinc-deficient endothelial cells causes a complete restoration of the endothelial cell barrier, whereas, this is not achieved with supplementation of calcium and magnesium [50]. Disturbances in the mineral balance of the vascular wall, which are often associated with disturbances in lipid metabolism, are regarded as significant factors in the progression of arteriosclerosis [24]. A protective effect of zinc has also been demonstrated for damage to the vascular wall caused by fatty acids [44,50]. In experimental studies on endothelial cells, Cornell et al. [51] found that zinc reduces TNF-α mediated activation of oxidative stress transcription factors, thereby reducing the increased synthesis of inflammatory cytokines and ultimately endothelial dysfunction. The endothelial protective effects of zinc include membrane-stabilising and antioxidant properties, the inhibition of essential steps in the cascades of both inflammatory reactions and apoptosis [24]. This means that when these protective functions fail due to zinc deficiency, oxidative stress and cell and tissue damage are increased. In addition, environmental influences and other stress factors can also have a damaging effect.

Besides these effects of zinc in the pathogenesis of arteriosclerosis, influences on endothelial signalling processes, effects in caspase-mediated apoptosis, a key position in endothelial Nitric oxide (NO) synthase activity and NO signalling are discussed [52,53]. Nakamura et al. [54] found in experimental studies an enlargement of pressure ulcers due to zinc deficiency. They concluded that zinc deficiency leads to vascular damage of the skin as a result of increased oxidative stress, forced apoptosis and an increase in ATP. With zinc supplementation, skin damage improved.

Abnormal accumulation of zinc in the brain is found in various neurological diseases, such as craniocerebral trauma, stroke and seizure disorders [8]. In the animal model with transient total and forebrain ischemia, an accumulation of zinc was observed as the last step of an ischemic insult before neuronal cell death [8]. Mammadova-Bach et al. [8] see a stroke as a thrombo-inflammatory event in which platelets and immune cells contribute to the extent of the ischemic vascular changes. Damage to the permeability of the blood-brain barrier triggers the death of neuronal cells. As a result of an ischemic stroke, zinc (Zn^2+^) is released into the synaptic cleft together with glutamate, an excitatory neurotransmitter, which leads to a sharp increase in zinc concentrations [8].

Numerous conventional factors such as arterial hypertension, diabetes mellitus, lipid metabolism disorders and genetic causes are involved in the etiopathogenesis of stroke [55]. According to Karadas et al. [55], changes in trace elements and heavy metals can affect acute haemorrhagic stroke. The authors found significantly lower serum zinc levels in patients with a haemorrhagic stroke than in controls without a stroke [55]. Similarly, Munhsi et al. [56] reported that serum zinc concentrations were also lower in stroke patients than in healthy controls, whereas no significant differences were found in copper and iron. The role of the zinc/copper ratio in the pathogenesis of haemorrhagic stroke has not yet been clearly clarified. Zhang et al. [57] state that zinc and copper compete for binding sites in cell membranes and that normal zinc and lower copper concentrations together reduce oxidative vascular damage and the associated risk of haemorrhagic stroke. In recent studies in hypertensive stroke patients, these authors found a significant inverse association between plasma zinc and the first occurrence of haemorrhagic stroke, although this association was more pronounced in obese patients and low plasma copper. A haemorrhagic stroke is mainly caused by a rupture and pathological changes in small vessels. The authors consider zinc to be a modifiable risk factor for haemorrhagic stroke, particularly in relation to the incidence of zinc deficiency in obesity and type 2 diabetes mellitus [57,58,59].

Close relationships also exist between the blood-brain barrier (BBB) and zinc. While the BBB is of great importance for maintaining zinc homeostasis in the brain, a proper balance between the zinc in the systemic circulation and in the brain is important for normal BBB function [60]. A disturbance of the zinc BBB system affects the microenvironment in the brain, which can lead to pathological damage. Qi et al. [60] conclude that zinc could serve as a potential target for protecting the BBB in stroke patients and reducing haemorrhagic transformation, inflammation and oedema.

In a recent review, Morais et al. [61] describe the close relationships between cortisol, insulin resistance (type 2 diabetes mellitus), zinc and obesity. Cortisol is a hormone and an important regulator of endocrine and metabolic functions. It contributes to fat accumulation in visceral fat stores and influences the metabolism of trace elements, especially of zinc. By activating the gene expression of metallothionein and zinc transporter ZIP 14, zinc is redistributed from the plasma to different tissues, especially to the liver and visceral fat, leading to hypozincaemia in obesity [61,62].

There are close interactions between zinc and obesity [63]. Various studies have shown significantly reduced blood concentrations of zinc in obese children and adults [63,64,65]. In these patients, low zinc levels are associated with an increase in metabolic disorders such as insulin resistance, inflammation and fat metabolism [63,65]. Zinc administration in overweight patients leads to an improvement of the body–mass index (BMI) and the lipid profile, especially the triglycerides (TG) [57]. Recently, Khorsadi et al. [58] report on the results of a randomised, placebo-controlled double-blind study in 40 obese patients. One group of 20 patients each received a zinc supplement (30 mg/day) or placebo for 15 weeks combined with a restricted diet of 300 kcal. The results show a favourable effect of zinc administration in combination with the restriction diet on weight, insulin resistance, inflammatory markers and appetite of the patients. According to studies by Iso et al. [66], a reduced intake of saturated fatty acids and animal protein is associated with an increased risk of parenchymal stroke. Various clinical observations and experimental studies show that reduced absorption of saturated and trans-unsaturated fat reduces platelet aggregability, which may lead to an increased risk of bleeding in the presence of arterial necrosis. The authors conclude that reduced absorption of saturated fat and trans unsaturated fat can lead to low serum cholesterol levels and reduced platelet aggregation and thus to intraparenchymal haemorrhages [67,68]. Intraparenchymal haemorrhages are caused by a rupture of microaneurysms resulting from arterial necrosis (fibrinoid necrosis or hyalinosis) of small intravertebral penetrating arterioles [67].

In a recent retrospective analysis of 384 patients with acute subarachnoid haemorrhage, Arleth et al. [69] investigated the frequency of serum zinc deficiency. They found reduced serum zinc levels in 67% (*n* = 257) of all patients within the first seven days after the event. The zinc deficiency was associated with a more severe course of disease. The authors consider zinc deficiency to be an independent factor that has an unfavourable influence on the course of the disease.

In the last two decades the interest in homocysteine, a sulphur-containing amino acid with close connections to vitamins B2, B6 and B12, has increased significantly. Changes in diet (meat-based, vegetarian, vegan), which are associated with changes in the availability of these vitamins, consequently influence the concentrations of homocysteine in the blood [70]. According to clinical studies, homocysteinaemia with an increased risk of thromboembolic disease (stroke, heart attack or thrombosis of the peripheral veins) has increased [71]. Since two enzymes in homocysteine metabolism, methionine synthase (MS) and betaine homocysteine methyltransferase (BHMT) are zinc-dependent, a zinc deficiency is a major factor in the increase in homocysteine concentration. Jing et al. [72] were able to show in studies on rats that a zinc deficiency is associated with increased homocysteine concentrations and reduced mRNA concentrations in MS. Barbato et al. [71] demonstrated an interaction between metallothionein and homocysteine. They showed that homocysteine reacts with metallothionein and thus releases zinc in homocysteinaemia. This leads to an inhibition of the scavenger function of metallothionein with subsequent release of zinc, which is associated with a disturbance in redox homeostasis. The indiscriminate release of zinc could have a strong influence on zinc-dependent intracellular protein expression through homocysteine [71] and may ultimately lead to a secondary, “non-food-related” zinc deficiency [27].

## 7. Conclusions

The increased incidence of haemorrhagic strokes in vegetarians or vegans compared to meat-eaters suggests that these diets are not well balanced, particularly with regard to essential nutrients, in the absence of a specific dietary composition.

**Figure 1:**
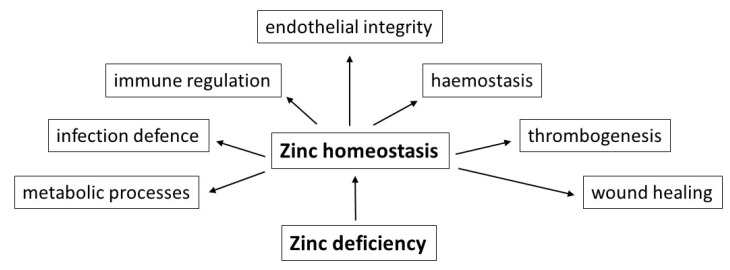
Schematic representation of important functional effects of a balanced zinc homeostasis in the context of food-induced zinc deficiency as possible risk factor for the development of haemorrhagic strokes.

In addition to the above-mentioned vital nutrients with low concentrations, such as vitamin B12, vitamin D, essential amino acids and long-chain *n*-3 polyunsaturated fatty acids, according to the authors’ many years of experience, deficiencies of trace elements, especially of zinc, should be taken also into account as a causative factor. Due to its essential role in numerous metabolic processes, in immune regulation and infection defence, in haemostasis and thrombogenesis, endothelial integrity and last but not least in the wound healing process, zinc deficiency could be a risk factor for the development of haemorrhagic strokes (Figure 1).

Future experimental and clinical studies should investigate the role of trace elements, especially zinc, in the genesis of haemorrhagic strokes. The possible connections that have been shown, which are certainly not exhaustive, should be the reason for further studies to clarify the situation regarding the frequency and epidemiological significance of strokes. In risk groups for the occurrence of zinc deficiency, such as patients > 65 years of age, with diabetes mellitus, obesity but also chronic liver or kidney diseases or rheumatoid arthritis, the zinc levels in serum or plasma should be checked. If a reproducible zinc deficiency is detected, controlled zinc substitution should be carried out.
